# Assessing the Relationship between Surgical Timing and Postoperative Seizure Outcomes in Cavernoma-Related Epilepsy: A Single-Institution Retrospective Analysis of 63 Patients with a Review of the Literature

**DOI:** 10.3390/brainsci14050494

**Published:** 2024-05-13

**Authors:** Elsa Nico, Christopher O. Adereti, Ashia M. Hackett, Andrea Bianconi, Anant Naik, Adam T. Eberle, Pere J. Cifre Serra, Stefan W. Koester, Samuel L. Malnik, Brandon M. Fox, Joelle N. Hartke, Ethan A. Winkler, Joshua S. Catapano, Michael T. Lawton

**Affiliations:** 1 Department of Neurosurgery, Barrow Neurological Institute, St. Joseph’s Hospital and Medical Center, Phoenix, AZ 85013, USA; 2 Department of Neurosurgery, Lahey Hospital and Medical Center, Burlington, MA 01805, USA

**Keywords:** antiepileptic drugs, cavernoma, cavernoma-related epilepsy, cavernous angioma, cavernous hemangioma, cavernous malformation, seizures, seizure outcomes

## Abstract

**Background**: Patients with supratentorial cavernous malformations (SCMs) commonly present with seizures. First-line treatments for cavernoma-related epilepsy (CRE) include conservative management (antiepileptic drugs (AEDs)) and surgery. We compared seizure outcomes of CRE patients after early (≤6 months) vs. delayed (>6 months) surgery. **Methods**: We compared outcomes of CRE patients with SCMs surgically treated at our large-volume cerebrovascular center (1 January 2010–31 July 2020). Patients with 1 sporadic SCM and ≥1-year follow-up were included. Primary outcomes were International League Against Epilepsy (ILAE) class 1 seizure freedom and AED independence. **Results**: Of 63 CRE patients (26 women, 37 men; mean ± SD age, 36.1 ± 14.6 years), 48 (76%) vs. 15 (24%) underwent early (mean ± SD, 2.1 ± 1.7 months) vs. delayed (mean ± SD, 6.2 ± 7.1 years) surgery. Most (32 (67%)) with early surgery presented after 1 seizure; all with delayed surgery had ≥2 seizures. Seven (47%) with delayed surgery had drug-resistant epilepsy. At follow-up (mean ± SD, 5.4 ± 3.3 years), CRE patients with early surgery were more likely to have ILAE class 1 seizure freedom and AED independence than those with delayed surgery (92% (44/48) vs. 53% (8/15), *p* = 0.002; and 65% (31/48) vs. 33% (5/15), *p* = 0.03, respectively). **Conclusions**: Early CRE surgery demonstrated better seizure outcomes than delayed surgery. Multicenter prospective studies are needed to validate these findings.

## 1. Introduction

Supratentorial cavernous malformations (SCMs) represent 65% to 80% of cerebral cavernous malformations (CCMs) and are characterized by abnormally dilated, thin-walled blood vessels lacking intervening parenchyma [1,2,3,4]. Given their low-flow circulation, angiographically occult SCMs are often identified incidentally using magnetic resonance imaging (MRI) or after symptomatic manifestations of hemorrhage, focal neurologic deficits, headache, and seizures [4,5,6,7]. Seizures are the most common presenting symptom of an SCM, accounting for 40% to 70% of initial symptoms, making SCMs the most epileptogenic cerebrovascular disease [8,9]. Although the annual seizure risk is only 2.4% per patient-year, the recurrent seizure risk is 5.5% per patient-year or up to 94% within 5 years [2,7,10].

Given the high seizure burden associated with SCMs, international guidelines recommend conservative management with an antiepileptic drug (AED) or microsurgical resection as first-line treatment options for new-onset cavernoma-related epilepsy (CRE) [11]. Although most patients with CRE are initially managed conservatively, AEDs harbor unwanted adverse effects, and up to 40% of patients develop drug-resistant epilepsy (DRE) [9,12]. However, numerous studies have demonstrated seizure freedom in approximately 75% of CRE patients after surgical intervention as a definitive treatment [11,13,14,15,16,17,18,19,20,21]. Despite the overwhelming evidence of seizure control efficacy, surgery is often reserved only for patients with refractory status epilepticus or DRE [11].

A longer duration of CRE before surgery has been associated with unfavorable postoperative seizure outcomes and AED dependency [13,14,15,18,19,20,22,23,24,25,26,27]. Only 1 study has intentionally assessed long-term outcomes stratified by surgical timing [21]. However, that study included patients who were conservatively managed in the overall cohort, and it distinguished early surgery from delayed surgery as <6 months vs. >12 months after CRE onset, thereby missing the period from 6 to 12 months [21]. To enhance the current understanding of the relationship between preoperative CRE duration and postoperative seizure outcomes, we compared rates of seizure freedom (International League Against Epilepsy [ILAE] class 1) and AED independence at follow-up in patients with CRE who underwent early surgery or delayed surgery.

## 2. Materials and Methods

### 2.1. Patient Selection

A database of patients with surgically treated SCMs was queried retrospectively at our large-volume cerebrovascular center to identify all patients who presented with 1 sporadic SCM and definite or probable CRE from 1 January 2010 to 31 July 2020. Definite CRE was defined as epilepsy in patients with an SCM and electroencephalographic evidence of seizure onset near the SCM; probable CRE was defined as epilepsy in patients with an SCM and electroencephalographic evidence of seizure onset in at least the same hemisphere as the SCM [11]. Additional eligibility criteria included preoperative electroencephalography recordings in the epilepsy monitoring unit (EMU) and MRIs, histopathologic diagnostic confirmation of an SCM, and follow-up of at least 1 year. Patients with a prior SCM surgical resection or recurrence were excluded from the analysis. Patients were categorized into 2 groups according to surgical timing relative to the preoperative duration of their CRE: early surgery (≤6 months) and delayed surgery (>6 months).

The institutional review board at St. Joseph’s Hospital and Medical Center (Phoenix, Arizona) approved the study. The board waived informed patient consent to publish because of the retrospective nature of the study and the low likelihood of patient identification.

### 2.2. Patient Demographics, Clinical Characteristics, and SCM Features

Patient charts were reviewed to extract demographic information (age, sex, and race or ethnicity) and clinical characteristics (smoking status, Charlson Comorbidity Index (CCI), functional status, preoperative CRE duration, number of preoperative seizures, and number and type of preoperative AEDs). The CCI predicts 10-year survival in patients with multiple comorbidities. Functional status was determined using the modified Rankin Scale (mRS) and categorized as favorable (mRS score ≤ 2) or unfavorable (mRS score > 2). In the delayed surgery group, DRE was defined as persistent seizures occurring for at least 2 years while taking at least 2 different AEDs; chronic epilepsy was defined as persistent seizures without qualifying as DRE [28]. Preoperative MRIs were evaluated for SCM features, including SCM lobar location (frontal, occipital, parietal, or temporal), cortical involvement, volume, and associated hemorrhage.

### 2.3. Outcomes

Primary outcomes were seizure freedom (ILAE class 1 [29]) and AED independence at follow-up. Patients who discontinued or never started AED therapy were classified as AED-independent at follow-up. Secondary outcomes included length of hospital stay, discharge disposition, the number and type of AEDs, favorable functional status, and recurrence and reoperation rate at follow-up. A favorable functional status at follow-up was defined as an mRS score ≤ 2.

### 2.4. Statistical Analysis

All statistical analyses were performed with R, version 4.0.1 (R Foundation for Statistical Computing). Categorical variables were analyzed using the chi-square test, and continuous variables were assessed using the Welch 2-sample *t*-test for parametric distributions and the Mann–Whitney test for nonparametric analyses. Statistical significance was defined as *p* < 0.05. Results are reported as mean ± SD and number (percentage).

### 2.5. Literature Review

The PubMed/MEDLINE (National Library of Medicine) database was queried in April 2023 for articles published in or translated into English using the terms “cavernous malformation” OR “cavernoma,” in combination with “seizure” OR “epilepsy” and “surgery,” “surgical,” or “outcome.” These search terms were used to investigate the relationship between preoperative CRE duration and postoperative seizure outcomes and AED independence. No restrictions on publication date were applied.

The articles retrieved after the initial search were screened for relevance to the research question. A thorough review of the abstracts of each article was conducted to identify the main findings and assess their relevance to the research question. The selected articles provided insights into the role preoperative CRE duration plays in postoperative seizure outcomes and AED independence.

## 3. Results

### 3.1. Patient Demographics, Clinical Characteristics, and SCM Features

**Figure 1:**
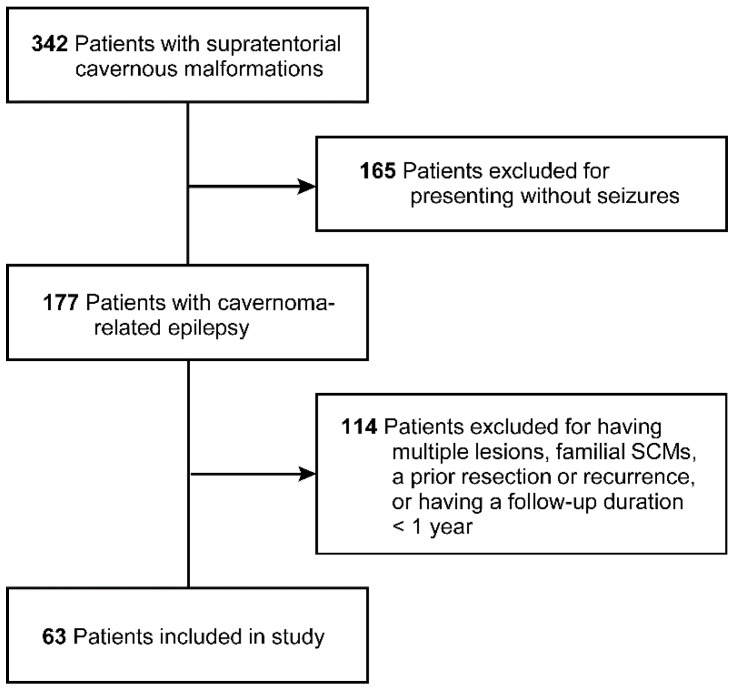
Patient selection flow diagram. *Used with permission from Barrow Neurological Institute, Phoenix, Arizona*.

The initial sample size included 342 patients with SCMs, of which 165 were excluded for presenting without seizures and 114 for having multiple lesions, familial SCMs, a prior resection or recurrence, or a follow-up duration < 1 year (Figure 1). A total of 63 patients with CRE were included in the final analysis, of whom 48 (76%) underwent early surgery (≤6 months) and 15 (24%) underwent delayed surgery (>6 months). The mean ± SD age at presentation was 36.1 ± 14.6 years; 37 (59%) were men, 26 (41%) were women, and most (49 (78%)) were white (Table 1). The frontal lobe was involved most often (25 (40%)), followed by the temporal (22 (35%)), parietal (12 (19%)), and occipital (4 (6%)). Most patients had cortical involvement (59 (94%)), a preoperative life expectancy of > 10 years (47 (75%)), and a favorable preoperative functional status (62 (98%)). The mean ± SD preoperative CRE duration was 19.5 ± 51.1 months (range, 0–348). Preoperative seizure frequency was split almost evenly between 1 seizure (32 (51%)) and 2 or more seizures (31 (49%)). Forty-six (73%) patients were taking an AED preoperatively, with most taking a single AED (38 (83%)), primarily levetiracetam (23/38 (61%)), rather than other AEDs such as lacosamide, lamotrigine, valproic acid, and phenytoin.

#### Early vs. Delayed Surgery

All demographic and SCM characteristics were comparable between the early surgery and the delayed surgery groups (*p* > 0.05), except for lobar location (Table 2). Temporal lobe localization was disproportionately present in the delayed surgery group relative to the early surgery group (10/15 (67%) vs. 12/48 (25%), *p* = 0.03). Although there was a predetermined 6-month cutoff for defining the 2 groups based on surgical timing, a wider gap in mean ± SD preoperative CRE duration was observed between the early surgery and delayed surgery groups (2.1 ± 1.7 vs. 74.9 ± 85.1 months, respectively; *p* < 0.001). Most (67% (32/48)) patients in the early surgery group had only 1 preoperative seizure, whereas all (15/15) in the delayed surgery group had 2 or more preoperative seizures (*p* < 0.001). Furthermore, all patients in the delayed surgery group received AED treatment preoperatively compared to only 65% (31/48) in the early surgery group (*p* = 0.02). In the delayed surgery group, 7 (47%) patients had DRE, and 8 (53%) had chronic epilepsy.

### 3.2. Outcomes

The mean ± SD duration of follow-up was 5.4 ± 3.3 years. At follow-up, most (83% (52/63)) patients had achieved or sustained ILAE class 1 seizure freedom, and 57% (36/63) had achieved or sustained AED independence (Table 1). Of the 27 remaining patients on AEDs at follow-up, fewer were taking levetiracetam (13 (48%)) than other AEDs (17 (63%)), and most (17 (63%)) were still taking a single AED. The mean length of stay was 2.8 ± 2.0 days, and of the 57 patients with information documented on discharge disposition, most (55 (96%)) were discharged home. At follow-up, all patients had a favorable functional status, no recurrence, and no reoperation.

#### Early vs. Delayed Surgery

A greater proportion of patients in the early surgery group demonstrated ILAE class 1 seizure freedom at follow-up than in the delayed surgery group (44/48 (92%) vs. 8/15 (53%), respectively; *p* = 0.002) (Table 2). Similarly, significantly more patients in the early surgery group than in the delayed group were AED-independent at follow-up (31 (65%) vs. 5 (33%), *p* = 0.03). Of note, 17 of the 31 patients in the early group who were AED-independent at follow-up were not on AEDs preoperatively. Thus, AED withdrawal rates at follow-up were comparable between the early and delayed surgery groups (14/31 (45%) vs. 5/15 (33%), *p* = 0.45). Despite no significant difference in the number of patients taking AEDs at follow-up between the early surgery and delayed surgery groups (17 (35%) vs. 10 (67%), respectively; *p* = 0.07), more patients in the delayed surgery group were taking an AED other than levetiracetam at follow-up (9 (60%) vs. 7 (17%), *p* = 0.003), and more patients were taking 2 or more AEDs (6 (40%) vs. 4 (8%), *p* = 0.01) than in the early surgery group.

### 3.3. Literature Selection

**Figure 2:**
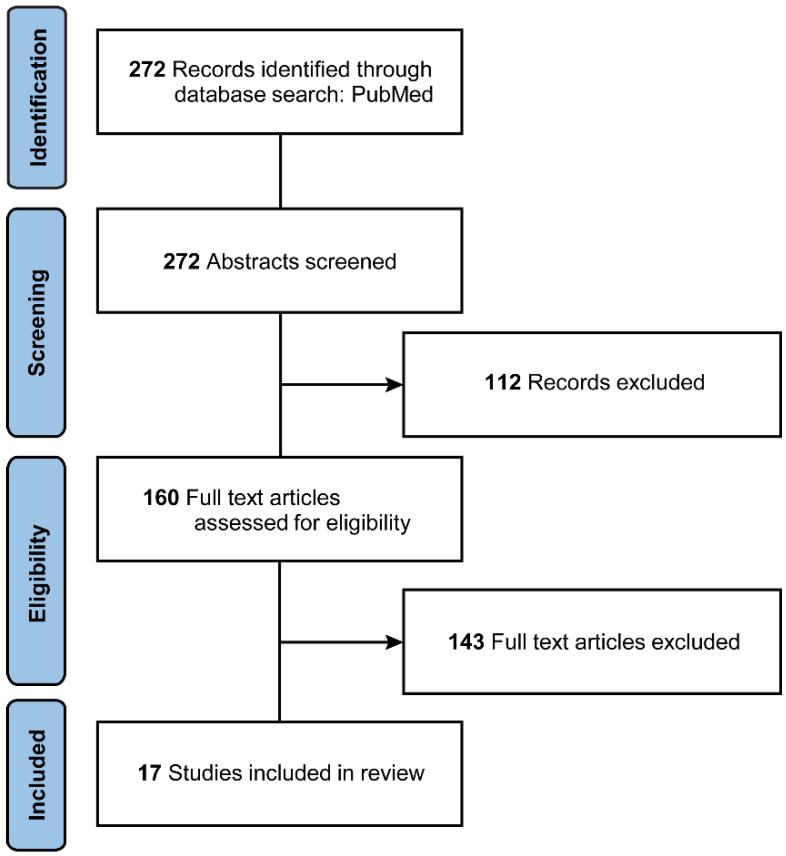
Literature selection flow diagram. *Used with permission from Barrow Neurological Institute, Phoenix, Arizona*.

A total of 272 articles were identified from the database during the initial search. After excluding non-English and nonhuman subject articles, as well as commentaries, editorials, letters to the editor, and reviews, the literature review yielded a total of 160 articles. The full-text screening for eligibility and availability for data extraction yielded 17 articles for final analysis (Figure 2). These articles were abstracted for key findings on demographics, preoperative CRE duration, seizure outcomes, and AED outcomes to compare the findings of our series with findings reported in the literature (Table 3) [13,14,15,16,18,20,21,22,23,24,25,26,27,30,31,32,33].

## 4. Discussion

CCMs represent 1 of the most common cerebrovascular malformations, and patients with supratentorial involvement are at an increased risk of developing recurrent seizures with the subsequent need for AED treatment to achieve seizure freedom [23]. DRE will likely develop in nearly 40% of the patients in this population [19,34,35]. CRE is characterized by endothelial cell dysfunction with incomplete or nonfunctional tight junctions that result in the leakage of blood degradation products into the surrounding parenchyma, which causes local inflammation and irritation [36]. Moreover, epileptogenicity is believed to arise from microhemorrhages and the resulting outer hemosiderin rim that triggers gliotic scarring and perifocal neurotic reactions [36]. However, the risk factors that contribute to CRE remain inconclusive and thus perpetuate ongoing discussions about the optimal management of patients with this condition [16,37,38].

Currently, no universally accepted treatment algorithm exists to manage CRE; hence, continued research is needed to provide greater clarity on the treatment of this condition [31,39]. Although many prior studies have addressed CRE treatment, we believe that our study is among the first to evaluate the temporal relationship between surgical intervention and postoperative CRE outcomes by establishing a 6-month CRE cutoff duration [13,14,15,18,19,20,22,23,24,25,26,27]. We determined that CRE patients who undergo early surgical intervention (≤6 months) achieve or sustain higher rates of ILAE class 1 seizure freedom and AED independence than CRE patients who undergo delayed surgery (>6 months) (92% vs. 53%, *p* = 0.002 and 65% vs. 33%, *p* = 0.03, respectively) at follow-up (mean ± SD, 5.4 ± 3.3 years).

### 4.1. Seizure Freedom

In this analysis, 83% of the 63 patients with CRE (mean ± SD, 19.2 ± 51.6 months) reached ILAE class 1 seizure freedom at a mean follow-up of 5.4 ± 3.3 years. Other studies with comparable cohort sizes and follow-up durations have identified similar seizure outcomes [21,25]. Zevgaridis et al. [25] found an 88% seizure freedom rate at a mean follow-up of 3.25 years in a 1996 study of 77 SCM patients with surgically treated CRE. Similarly, in a 2017 analysis, Dammann et al. [21] showed that 85% of 60 patients with CRE who underwent immediate (n = 41) or delayed surgery (n = 19) achieved a minimum of 2 years ILAE class 1 continuous seizure freedom at the last follow-up. In contrast to these findings, a large multicenter study by Baumann et al. [16] (n = 168 SCMs with CRE) found that 70%, 68%, and 65% of patients had an Engel class 1 seizure outcome after 1, 2, and 3 years, respectively. Of note, these patients had a longer mean preoperative CRE duration of 8 years than those in our study. Likewise, lower rates of seizure freedom were described by Stefan et al. [30] (n = 30 SCMs with CRE) and Folkersma and Mooij [40] (n = 7 SCMs with DRE) of 53% and 57%, respectively; however, these studies had smaller cohorts. The results of these studies contrast with those of other series, some with lower preoperative seizure frequencies and shorter preoperative CRE durations, which showed 78% to 100% seizure freedom rates [13,18,20,22,23,24,31,33,41].

#### Early vs. Delayed Surgery

Among our patients with CRE, those who underwent early surgery (n = 48), with a mean preoperative CRE duration of 2.1 ± 1.7 months, were more likely to achieve or sustain higher rates of ILAE class 1 seizure freedom than those who underwent delayed surgery (n = 15), with a mean preoperative CRE duration of 6.2 ± 7.1 years (92% vs. 53%, respectively; *p* = 0.002). Our results are consistent with those of many observational studies that identified a negative correlation between a longer preoperative CRE duration and worse seizure outcomes (Table 3) [13,14,15,16,18,20,21,22,23,24,25,26,27,30,31,32,33]. Most authors report a significantly worse seizure outcome for patients with a preoperative CRE duration longer than 1 to 2 years, except for patients who have a long history of sporadic seizures (Table 3) [13,14,18,22,23,24,25,26]. In a 2023 study of 37 CREs, Shoubash et al. [33] reported that a longer mean preoperative CRE duration was significantly associated with unfavorable Engel class 2 to 4 seizure outcomes (174.2 ± 114.7 vs. 28.4 ± 97.0 months, respectively; *p* = 0.0043) compared to favorable Engel class 1 seizure outcomes. Surgery should be considered in patients presenting with a single seizure as a preventative measure given that up to 94% of patients have a recurrent seizure within 5 years [2,7,10].

Other authors, however, have found no association between preoperative CRE duration and seizure outcomes (Table 3) [16,21,27,31,32]. Dammann et al. [21] showed significantly improved ILAE class 1a seizure freedom and sustained 2-year ILAE class 1 seizure freedom outcomes at the last follow-up in CRE patients who underwent immediate surgery (n = 41), with a mean preoperative CRE duration of 2.6 ± 1.7 months rather than initial conservative treatment (n = 38) (73% vs. 24%, respectively, *p* < 0.0001; 88% vs. 32%, respectively, *p* < 0.0001). Similarly, patients who underwent delayed surgery (n = 19), with a mean preoperative CRE duration of 43 ± 30 months, improved more than patients who initially underwent conservative treatment (63% vs. 24%, respectively, *p* < 0.05; 79% vs. 32%, respectively, *p* < 0.0005). However, no significant difference was noted in seizure outcomes between the immediate and delayed surgery groups (73% vs. 63%, respectively, *p* = 0.5468; 88% vs. 79%, respectively, *p* = 0.4165) [21]. Notably, the comparable seizure outcomes between these immediate and delayed surgery groups are likely attributable to the smaller cohort and fewer complete follow-up visits in the delayed group, which limited the statistical analysis. In contrast to our results, Shoubash et al. [33] reported that a longer preoperative CRE duration predicted worse seizure outcomes but that a preoperative CRE duration of < 6 months vs. > 6 months produced no significant variation in outcomes.

### 4.2. AED Independence

In our cohort of 63 patients, 57% with CRE achieved or sustained AED independence at follow-up. Although data on postoperative AED usage are limited and highly variable, our follow-up AED independence rate falls between previously reported AED withdrawal rates [13,32,41]. Regarding lower AED withdrawal rates, Fernández et al. [41] noted “nonrefractory” CRE in 35% (9/26) and 33% (5/15) of patients at year 2 and year 5 of follow-up, respectively. Concerning higher rates of AED withdrawal at follow-up, Cappabianca et al. [13] showed a 63% AED withdrawal rate in 19 patients with a short CRE duration (< 12 months) and low seizure frequency of 1–5 seizures, whereas Ozlen et al. [32] found that 78% of drug-responsive CRE patients (n = 40) were weaned off AEDs compared to 69% of DRE patients (n = 16).

#### Early vs. Delayed Surgery

In our study, patients in the early surgery group achieved or sustained significantly higher rates of AED independence than those in the delayed surgery group (65% vs. 33%, *p* = 0.03). Only 2 studies in the medical literature have compared AED withdrawal rates in CRE patients stratified by surgical timing (Table 3) [21,23]. Similar to our findings, those of Kapadia et al. [23] indicated that 79% of patients who underwent early surgery (n = 19) were successfully weaned off AEDs at follow-up compared to only 25% of patients who underwent delayed surgery (n = 16) (*p* = 0.001). In contrast, despite reporting a high 72% AED withdrawal rate at the last follow-up overall, Dammann et al. [21] found no significant difference between patients who underwent immediate surgery (n = 41) vs. delayed surgery (n = 19) (78% vs. 58%, respectively; *p* > 0.05).

### 4.3. Choice of AED

Of the 38 patients in our study who took a single preoperative AED, 23 (61%) were taking levetiracetam compared to 15 (39%) taking a different AED. At follow-up, levetiracetam use had decreased by 11%, from 27/46 (59%) to 13/27 (48%), whereas other AED use had increased by 17%, from 21/46 (46%) to 17/27 (63%), in the overall cohort. When stratified by surgical groups, patients in the delayed surgery group were more likely to be taking a preoperative AED other than levetiracetam than patients in the early surgery group (11/15 (73%) vs. 10/31 (32%)), respectively; *p* = 0.001).

Furthermore, 9/10 (90%) of patients in the delayed surgery group remained on an AED other than levetiracetam at follow-up vs. 8/17 (47%) in the early surgery group (*p* = 0.003). Although guidelines supporting the use of levetiracetam as the first-line AED for CRE management are controversial [42,43], possible explanations for using this medication include its favorable safety profile, distinct mechanism of action, simple dosing schedule, fewer drug interactions, and tolerability in pregnancy [44]. Nevertheless, AEDs are associated with several adverse effects that can lead to patient misuse or low compliance and, therefore, higher rates of DRE and breakthrough seizures. The risk of developing DRE necessitates prospective studies to examine various AEDs and to clarify first-line AED recommendations for CRE.

### 4.4. SCM Lobar Location

A greater proportion of patients in the delayed surgery group than in the early surgery group had SCMs localized to the temporal lobe (67% vs. 25%, respectively; *p* = 0.03). Although this finding may explain why patients who undergo delayed surgery experience lower rates of seizure freedom, most studies indicate that temporal involvement does not predict seizure outcome [13,16,18,27,30]. Although Baumann et al. [16] reported that patients with neocortical temporal cavernomas had a greater risk of poor seizure outcome (Engel class 4) at the 1-year follow-up than those with mesiotemporal cavernomas (22% vs. 0%, respectively; *p* = 0.04), no such difference was noted at subsequent 2- and 3-year follow-up visits. Moreover, in a 2020 CRE study focusing specifically on SCMs localized in the temporal lobe, Schuss et al. [45] found that most patients (47/52 (90%)) achieved ILAE class 1 seizure freedom at 1 year of follow-up.

### 4.5. Benefits of Early Surgical Intervention

#### 4.5.1. Reduced Frequency and Number of Seizures

Over time, frequent seizure activity in patients with CRE may worsen the epileptogenic focus, with a resultant decrease in the rate of postoperative seizure freedom, which favors early surgery [13,14,15,18,22,23,27,30,33,46]. In a 2021 study assessing 35 CRE patients, Kapadia et al. [23] observed higher rates of seizure freedom among patients who had ≤ 2 preoperative seizures (n = 19) (median preoperative CRE duration of 2 (range, 1–24) months) relative to those with >2 preoperative seizures (n = 16) (median preoperative CRE duration of 24 (range, 1–239) months) of 95% vs. 62%, respectively, at 1-year follow-up (*p* = 0.019). This seizure outcome was consistent with long-term follow-up (median 5.6 years), whereby all patients experiencing ≤2 preoperative seizures remained seizure-free compared to 50% of patients who experienced >2 preoperative seizures [23]. Similarly, Yeon et al. [18] found that patients with sporadic seizures were more likely to achieve Engel class 1A seizure freedom at follow-up than patients with intractable epilepsy (>1 seizure/month over a year) (84% (32/38) vs. 55% (12/22), respectively; *p* = 0.034). Similarly, our analysis showed that 67% of patients in the early group experienced only 1 preoperative seizure compared to 100% of patients in the delayed group who experienced ≥ 2 preoperative seizures (*p* < 0.001), with better seizure outcomes in the former group than the latter [23]. However, few studies have shown no association between preoperative seizure frequency and seizure outcomes [16,17,24,38].

#### 4.5.2. Decreased Risk of Developing DRE

Another benefit of early surgical intervention is the decreased likelihood of developing DRE [23,32,47]. DRE develops in approximately 40% of patients with CRE [9,12,19,34,35]. In our study, 47% (7/15) of patients in the delayed surgery group had DRE compared to the 38% (6/16) with DRE reported by Kapadia et al. [23] Patients in the delayed surgery group were also more likely to take 2 or more AEDs at follow-up than patients in the early surgery group (40% vs. 8%, respectively; *p* = 0.01).

#### 4.5.3. Hemosiderin Deposits

Radiologic evidence of residual hemosiderin deposits on postoperative brain MRIs is significantly associated with poor seizure outcomes [21,34]. In 2017, Dammann et al. [21] reported MRI evidence of residual hemosiderin in 14 patients with available postoperative MRIs, and 10 had worse associated seizure outcomes (OR 38.75, 95% CI 6.14–244.23, *p* < 0.0001). Conversely, Menzler et al. [48] reported no correlation between the presence (OR 0.85, 95% CI 0.24–2.9, *p* = 0.79) or the diameter (OR 1.1, 95% CI 0.96–1.2, *p* = 0.23) of the hemosiderin rim (mean 4.0 ± 3.6 mm) and epilepsy in 85 patients. However, evidence suggests that early surgery should be pursued before hemosiderin deposits expand into the surrounding parenchyma because of recurrent hemorrhage risk [11,49]. Furthermore, given the bleeding risk and the negative association between preoperative CRE duration and postoperative seizure outcomes highlighted in previous studies, most authors do not recommend waiting for DRE to develop, as proposed by the ILAE, before offering surgical treatment to CRE patients [11,50]. Thus, surgery for CRE should be considered as an option after the failure of a single trial of an appropriate AED [11].

### 4.6. Limitations

Our study and the analysis of our results have several important limitations. First, the retrospective nature of this study limited the information gathered from the chart review. CRE patients with familial or multiple SCMs, prior surgical interventions, and follow-up appointments of less than 1 year were excluded, which reduced the sample size. However, these exclusions were intentional to limit the possibility of confounding that would have skewed outcomes. Of note, the 8 patients with hemorrhage were found only in the early surgery group, and it is unclear whether they were offered surgery to eliminate the risk of hemorrhage rather than to control seizures. Although the mean duration of follow-up for seizure and AED outcomes was 5.4 years, sustained seizure freedom after surgery or for at least 2 years was not assessed. According to Josephson et al. [10], sustained seizure freedom is a more clinically relevant seizure outcome than seizure freedom at a minimum 1-year follow-up because AEDs are rarely withdrawn before 2 years of sustained seizure freedom. Given that our definition of AED independence included patients who were never treated with AEDs, our AED independence rates might not be comparable to those reported by studies that assessed solely AED withdrawal. Furthermore, outcomes in patients with a single preoperative seizure were not compared with outcomes of patients with DRE and multiple preoperative seizures.

Additionally, our findings may not be widely generalizable, given that all patients were treated at a single institution by highly experienced neurosurgeons. Future research involving larger multicenter studies may help overcome this limitation. Lastly, outcomes were not stratified by seizure type, eloquent location, hemorrhage size, or associated developmental venous anomaly based on the reporting standards for CCM research [51]. Ongoing research evaluating surgical timing related to these factors may further underscore the importance of early surgical intervention for improving CRE outcomes.

## 5. Conclusions

Patients with CRE who underwent early surgery achieved or sustained higher rates of ILAE class 1 seizure freedom and AED independence than patients who underwent delayed surgery. Although multicenter prospective studies are needed to validate these findings, our results suggest that patients with CRE may benefit more from early surgery and have more favorable long-term outcomes than patients who undergo delayed surgery. Besides factors such as age, comorbidities, or SCM location, surgery could be considered for all patients with CRE to minimize dependency on AEDs and resistance to AEDs and to maximize patients’ quality of life.

## Figures and Tables

**Table 1:** Demographics, supratentorial cavernous malformation features, clinical characteristics, and outcomes of 63 patients with cavernoma-related epilepsy *.

Variable	Value
**Demographics**	
Age at presentation, mean ± SD, y	36.1 ± 14.6
Sex	
Male	37 (59)
Female	26 (41)
Race/Ethnicity †	
White	49 (78)
Black	3 (5)
Hispanic	6 (10)
Asian	2 (3)
Native American	3 (5)
**SCM features**	
Lobar location	
Frontal	25 (40)
Occipital	4 (6)
Parietal	12 (19)
Temporal	22 (35)
Right hemisphere	32 (51)
Cortical involvement	59 (94)
Lesion volume, mean ± SD, cm^3^	2.9 ± 3.8
Hemorrhage	8 (13)
**Clinical characteristics**	
Smoker	18 (29)
Former smoker	11/18 (61)
Current smoker	7/18 (39)
CCI score †	
0	47 (75)
1	3 (5)
2	8 (13)
3	5 (8)
Preop mRS score, mean ± SD	1.2 ± 0.5
Preop mRS score ≤2	62 (98)
Preop CRE duration, mean ± SD, mo	19.5 ± 51.1
Preop CRE duration	
≤6 mo	48 (76)
>6 mo	15 (24)
No. of preop seizures	
1	32 (51)
2	7 (11)
≥3	24 (38)
Preop AED	46 (73) §
Levetiracetam	27/46 (59)
Other	21/46 (46)
No. of preop AEDs	
1	38/46 (83)
Levetiracetam	23/38 (61)
Other	15/38 (39)
2	8/46 (17)
**Outcomes**	
Follow-up duration, mean ± SD, y	5.4 ± 3.3
Follow-up seizure freedom	52 (83)
Follow-up AED independence ‡	36 (57)
Follow-up AED	27 (43) §
Levetiracetam	13/27 (48)
Other	17/27 (63)
No. of follow-up AEDs	
1	17/27 (63)
2	9/27 (33)
3	1/27 (4)
Follow-up mRS score, mean ± SD	0.4 ± 0.6
Follow-up mRS score ≤ 2	63 (100)
LOS, mean ± SD, d	2.8 ± 2.0
Discharge disposition	
Home	55/57 (96)
Acute rehabilitation	2/57 (4)
Recurrence	0 (0)
Reoperation	0 (0)

* Data are presented as number (%) unless otherwise indicated. † Percentages total > 100% because of rounding. ‡ AED independence was defined as discontinuing or never starting AEDs. § Some patients took more than 1 AED. Abbreviations: AED = anti-epileptic drug; CCI = Charlson Comorbidity Index; CRE = cavernoma-related epilepsy; LOS = length of stay; mRS = modified Rankin Scale; preop = preoperative; SCM = supratentorial cavernous malformation.

**Table 2:** Early vs. delayed surgery in 63 patients with cavernoma-related epilepsy *.

Variable	Early Surgery (n = 48)	Delayed Surgery (n = 15)	*p* Value
**Demographics**			
Age at presentation, mean ± SD, y	34.2 ± 14.3	42.3 ± 14.6	0.06
Sex			0.43
Male	30 (62)	7 (47)	
Female	18 (38)	8 (53)	
Race/Ethnicity †			0.13
White	39 (81)	10 (67)	
Black	3 (6)	0 (0)	
Hispanic	3 (6)	3 (20)	
Asian	2 (4)	0 (0)	
Native American	1 (2)	2 (13)	
**SCM features**			
Lobar location			**0.03**
Frontal	22 (46)	3 (20)	
Occipital	4 (8)	0 (0)	
Parietal	10 (21)	2 (13)	
Temporal	12 (25)	10 (67)	
Right hemisphere	26 (54)	6 (40)	0.51
Cortical involvement	45 (94)	14 (93)	0.95
Lesion volume, mean ± SD, cm^3^	3.2 ± 4.1	1.8 ± 2.3	0.20
Hemorrhage	8 (17)	0 (0)	0.21
**Clinical characteristics**			
Smoker	11 (23)	7 (47)	0.15
Former smoker	6/11 (55)	5/7 (71)	0.14
Current smoker	5/11 (45)	2/7 (29)	>0.99
CCI †			0.16
0	38 (79)	9 (60)	
1	3 (6)	0 (0)	
2	4 (8)	4 (27)	
3	3 (6)	2 (13)	
Preop mRS score, mean ± SD	1.2 ± 0.6	1.3 ± 0.5	0.81
Preop mRS score ≤ 2	47 (98)	15 (100)	>0.99
Preop CRE duration, mean ± SD, mo	2.1 ± 1.7	74.9 ± 85.1	**<0.001**
No. of preop seizures			**<0.001**
1	32 (67)	0 (0)	
2	6 (12)	1 (7)	
≥3	10 (21)	14 (93)	
Preop AED	31 (65)	15 (100) §	**0.02**
Levetiracetam	21 (68)	6 (40)	>0.99
Other	10 (32)	11 (73)	**0.001**
No. of preop AEDs †			**<0.001**
0	17 (35)	0 (0)	
1	30 (62)	8 (53)	
Levetiracetam	20/30 (67)	3/8 (38)	0.27
Other	10/30 (33)	5/8 (62)	0.27
2	1 (2)	7 (47)	
**Outcomes**			
Follow-up duration, mean ± SD, y	5.4 ± 3.0	5.6 ± 4.0	0.83
Follow-up seizure freedom	44 (92)	8 (53)	**0.002**
Follow-up AED independence ‡	31 (65)	5 (33)	**0.03**
Follow-up AED	17 (35) §	10 (67)	0.07
Levetiracetam	11/17 (65)	2/10 (20)	0.66
Other	8/17 (47)	9/10 (90)	**0.003**
No. of follow-up AEDs			**0.02**
0	31 (65)	5 (33)	
1	13 (27)	4 (27)	
2	4 (8)	5 (33)	
3	0 (0)	1 (7)	
Follow-up mRS score, mean ± SD	0.4 ± 0.6	0.6 ± 0.6	0.22
Follow-up mRS score ≤ 2	48 (100)	15 (100)	
LOS, mean ± SD, d	2.8 ± 2.1	2.6 ± 1.7	0.82
Discharge disposition			>0.99
Home	44/46 (96)	11/11 (100)	
Acute rehabilitation	2/46 (4)	0/11 (0)	
Recurrence	0 (0)	0 (0)	
Reoperation	0 (0)	0 (0)	

* Data are presented as number (%) unless otherwise indicated. Boldfaced *p* values indicate statistical significance (*p* < 0.05). † Percentages may not total 100% because of rounding. ‡ AED independence was defined as discontinuing or never starting AED treatment. § Some patients took more than 1 AED. Abbreviations: AED = anti-epileptic drug; CCI = Charlson Comorbidity Index; CRE = cavernoma-related epilepsy; LOS = length of stay; mRS = modified Rankin Scale; preop = preoperative.

**Table 3:** Summary of studies correlating preoperative cavernoma-related epilepsy duration with postoperative seizure outcomes and anti-epileptic drug use outcomes *.

Author, Year [Ref.]	Surgical Cohort, No.	Age at Surgery, y	Sex, M/F, No. (%)	Preop CRE Duration, mo	Follow-Up Duration, mo	Seizure Outcomes	AED Outcomes
Cohen et al., 1995 [22] †	N = 50	34.7 ± 1.8	21/29 (42/58)	40.4 ± 10.8 (***p*** = **0.03**) 92.3 ± 29.8	59.8 ± 5.2	70% (35/50) seizure free; 30% (15/50) cont. seizures	57% (20/35) seizure free *w*/*o* AEDs; 14% (5/35) seizure free w/AEDs; 29% (10/35) seizure free w/AEDs or AED taper; 100% (15/15) cont. seizures w/AEDs
Casazza et al., 1996 [24] †	N = 47 (26 w/sporadic seizures; 21 w/chronic seizures)	32.4 ± 15.6	29/18 (62/38)	18 ± 22.8 122.4 ± 109.2	24	96% (25/26) seizure free w/AEDs; 86% (18/21) seizure free w/AEDs	NA
Zevgaridis et al., 1996 [25] †	N = 77 (47 w/<2y seizure history; 30 w/>2y seizure history)	36.8 (range, 3–72) ‡	36/41 (47/53)	58 (range, 0.25–600)	39	96% (45/47) seizure free; 77% (23/30) seizure free	62% (48/77) seizure free *w*/*o* AEDs
Cappabianca et al., 1997 [13] †	N = 35 (19 w/<5 preop seizures or <12-mo seizure history; 16 w/>5 preop seizures or >12-mo seizure history)	28.8 (range, 6 mo–74 y)	14/21 (40/60)	NA	24	100% (19/19) seizure free; 62% (10/16) seizure free	63% (12/19) seizure free *w*/*o* AEDs; 6% (1/16) seizure free *w*/*o* AEDs; 56% (9/16) seizure free w/AEDs; 38% (6/16) w/seizures w/AEDs
Moran et al., 1999 [14] †	N = 17	37.3 ± 8.5	8/9 (47/53)	144 ± 120 (*p* > 0.05) 252 ± 144	38.4 ± 25.2	35% (6/17) seizure free (Engel class 1); 24% (4/17) Engel class 2; 18% (3/17) Engel class 3; 24% (4/17) Engel class 4	NA
Stefan et al., 2004 [30] †	N = 30	39.4 (range, 19–62)	18/12 (60/40)	55.2 ± 74.28 217.2 ± 133.8	48	53% (16/30) seizure free (Engel class 1A or ILAE class 1); 47% (14/30) cont. seizures, 3% (1/30) auras (Engel class 1B or ILAE class 2); 27% (8/30) Engel class 2A–3B or ILAE class 3–5; 13% (4/30) Engel class 4A–4B or ILAE class 5; 3% (1/30) Engel class 4C or ILAE class 6	25% (4/16) seizure free *w*/*o* AEDs; 100% (14/14) cont. seizures w/AEDs
Ferroli et al., 2006 [15] †	N = 163 (64 w/single or sporadic seizures; 99 w/longer history)	33.4 ± 14.2	NA	14.4 ± 20.4 122.4 ± 109.2	48	98% (63/64) seizure free; 69% (68/99) seizure free	84% (53/63) seizure free *w*/*o* AEDs; 71% (48/68) seizure free *w*/*o* AEDs
Baumann et al., 2007 [16]	N = 168	30 ± 15	90/78 (54/46)	96 ± 108	25 ± 6	1-y follow-up: 70% (118/168) Engel class 1; 18% (31/168) Engel class 2; 6% (10/168) Engel class 3; 5% (9/168) Engel class 4. 2-y follow-up: 68% (83/122) Engel class 1; 18% (22/122) Engel class 2; 7% (8/122) Engel class 3; 7% (9/122) Engel class 4. 3-y follow-up: 65% (64/99) Engel class 1; 18% (18/99) Engel class 2; 8% (8/99) Engel class 3; 9% (9/99) Engel class 4	1-y follow-up: 95% (160/168) w/AEDs; 2-y follow-up: 85% (104/122) w/AEDs; 3-y follow-up: 76% (75/99) w/AEDs
Hammen et al., 2007 [26] †	N = 30	39.4 ± 12.4	17/13 (57/43)	55.2 217.2	≤48	53% (16/30) seizure free (Engel class 1A or ILAE class 1); 47% (14/30) cont. seizures (Engel class 1B–4B or ILAE class 2–5)	NA
Yeon et al., 2009 [18] †	N = 60 (38 w/sporadic seizures; 22 w/intractable epilepsy)	28.4 ± 12.5 ‡ 25.3 ± 11.9 ‡	37/23 (62/38)	40.8 (range, 1.2–408) 88.8 (range, 12–312)	31.2 ± 21.6 41.4 ± 23.6	89% (34/38) seizure free (Engel class 1); 73% (16/22) seizure free (Engel class 1)	77% (41/53) seizure free *w*/*o* AEDs
Kivelev et al., 2011 [27]	N = 40	36 (range, 10–60) §	11/29 (27/73)	36 (range, 1.2–276)	72 (range, 2.4–312) ¶	100% (10/10) seizure free w/1 preop seizure; 69% (11/16) seizure free w/2–5 preop seizures; 60% (9/15) seizure free w/numerous preop seizures	38% (15/39) seizure free *w*/*o* AEDs or w/AED taper
von der Brelie et al., 2013 [20] †	N = 118 (22 w/sporadic seizures; 20 w/chronic epilepsy; 76 w/drug-resistant epilepsy)	36 40 40	71/47 (60/40) 10/10 (50/50) 48/28 (63/37)	3.6 (***p*** = **0.01**) 39.6 198	111 ± 53.3 141 ± 64.4 134 ± 63	91% (20/22) seizure free (ILAE class 1); 80% (16/20) seizure free (ILAE class 1); 88% (67/76) seizure free (ILAE class 1)	NA
Dammann et al., 2017 [21]	N = 60 (41 w/initial surgery; 19 w/delayed surgery)	39 36	22/17 (56/44) 20/16 (53/47)	2.6 ± 1.7 43 ± 30	69 ± 34 56 ± 31	73% (30/41) seizure free (ILAE class 1a) (*p* = 0.5468); 63% (12/19) seizure free (ILAE class 1a); 88% (36/41) seizure free at 2 y (ILAE class 1) (*p* = 0.4165); 79% (15/19) seizure free at 2 y (ILAE class 1)	78% (32/41) seizure free *w*/*o* AEDs (*p* > 0.05); 58% (11/19) seizure free *w*/*o* AEDs
Kapadia et al., 2021 [23] †	N = 35 (19 w/ ≤2 preop seizures; 16 w/ >2 preop)	40.9 (range, 27–57), 46.1 (range, 20–73)	12/7 (63/37) 8/8 (50/50)	2 (range, 1–24) (***p* < 0.001**) 24 (range, 1–239)	67.2 (range, 12–132) 51.6 (range, 12–108)	95% (18/19) seizure free at 1-y follow-up (***p*** = **0.019**); 62% (10/16) seizure free at 1-y follow-up	79% (15/19) seizure free *w*/*o* AEDs (***p*** = **0.001**); 25% (4/16) seizure free *w*/*o* AEDs
Dziedzic et al., 2022 [31]	N = 45	34.6 (range, 19–70) 32.9 (range, 17–52)	17/18 (49/51) 6/4 (60/40)	55.4 ± 75.1 (*p* = 0.382) 53 ± 114.1	44.8 (range, 12–161.8) 43.8 (range, 21.6–112.9)	78% (35/45) seizure free (Engel class 1); 22% (10/45) cont. seizures (Engel class 2–4)	NA
Ozlen et al., 2022 [32]	N = 56 (40 w/drug-responsive CRE; 16 w/drug-resistant CRE)	32.3 (range, 11–54) 28.3 (8–45)	19/21 (48/52) 7/9 (44/56)	17.5 (range, 1–57) (***p* < 0.001**) 36.1 (range, 13–82)	69.6 (range, 24–216)	100% (40/40) seizure free (Engel class 1) (***p* < 0.01**); 81% (13/16) seizure free (Engel class 1)	78% (31/40) seizure free *w*/*o* AEDs; 15% (6/40) seizure free w/1 AED; 8% (3/40) seizure free w/AEDs; 69% (11/16) seizure free *w*/*o* AEDs; 12% (2/16) w/1 AED; 19% (3/16) w/AEDs
Shoubash et al., 2023 [33] †	N = 37	36.5 ± 14.1 50 ± 11.8	22/15 (59/41)	28.4 ± 97 (***p* = 0.0043**) 174.2 ± 114.7	67.2 ± 46.8	86% (32/37) seizure free (Engel class 1–1a); 14% (5/37) cont. seizures (Engel class 2–4)	NA
Present study, 2023 †	N = 63 (48 w/early surgery; 15 w/delayed surgery)	34.2 ± 14.3 42.3 ± 14.6	30/18 (63/37) 7/8 (47/53)	2.1 ± 1.7 (***p* < 0.001**) 74.9 ± 85.1	64.8 ± 36 67.2 ± 48	92% (44/48) seizure free (***p* = 0.002**); 53% (8/15) seizure free	65% (31/48) *w*/*o* AEDs (*p* = 0.03); 33% (5/15) *w*/*o* AEDs

* Values are mean ± SD, mean (range), or number (%) unless otherwise indicated. Percentages may total ≤100% or ≥100% because of rounding. Boldfaced *p* values indicate statistical significance (*p* < 0.05). † Article indicates longer preoperative CRE durations are associated with worse seizure outcomes. ‡ Age at onset of CRE. § Median age at diagnosis. ¶ Median follow-up. Abbreviations: AEDs = anti-epileptic drugs; cont. = continued; CRE = cavernoma-related epilepsy; F = female; M = male; NA = not applicable; preop = preoperative; Ref. = reference; w/ = with; *w*/*o* = without.

## Data Availability

The data supporting this study’s findings are not openly available but are available from the corresponding author upon reasonable request. The data are not publicly available because they are in controlled-access (HIPAA compliant) data storage at Barrow Neurological Institute.

## Abbreviations

AED	antiepileptic drug
CCM	cerebral cavernous malformation
CRE	cavernoma-related epilepsy
DRE	drug-resistant epilepsy
EMU	epilepsy monitoring unit
ILAE	International League Against Epilepsy
MRI	magnetic resonance imaging
mRS	modified Rankin Scale
SCM	supratentorial cavernous malformation
